# A Bioinformatic Investigation of the Mechanism Underlying Migraine-Induced Erectile Dysfunction

**DOI:** 10.1155/2021/6674643

**Published:** 2021-05-03

**Authors:** Ji-sheng Wang, Sheng Deng, Qi Zhao, Kai-ge Zhang, Bing-hao Bao, Jun-long Feng, Fan-chao Meng, Heng-heng Dai, Xiao Li, Hai-song Li, Bin Wang

**Affiliations:** ^1^First Clinical Medical College, Beijing University of Chinese Medicine, Beijing, BJ 100029, China; ^2^Andrology Department, Dongzhimen Hospital, Beijing University of Chinese Medicine, Beijing, BJ 100700, China; ^3^Department of Encephalopathy, Dongzhimen Hospital, Beijing University of Chinese Medicine, Beijing, BJ 100700, China; ^4^The First Affiliated Hospital, Henan University of Chinese Medicine, Zhengzhou 450000, China

## Abstract

**Background:**

Over recent years, an increasing body of literature has focused on the relationship between erectile dysfunction (ED) and migraine. However, the specific mechanism is unclear.

**Materials and Methods:**

We used a bioinformatic database to predict the targets and pathways associated with migraine and ED. Twenty male SD rats were randomly divided into a blank group (Group A, *n* = 10) and a migraine model group (Group B, *n* = 10). The rats in Group A were subcutaneously injected with normal saline (2 ml/kg) into the back of the neck. Rats in Group B were subcutaneously injected with nitroglycerin 10 mg/kg (5 mg/ml) into the back of the neck in order to create an animal model of migraine. Next, we carried out the measurement of erectile function. We used hematoxylin and eosin (HE) to compare the tissue structure of the cavernous body of the penis. Western blotting was used to determine the expression levels of PI3K, p-AKT, and p-mTOR in the protein; Reverse Transcription-Polymerase Chain Reaction (RT-qPCR) was used to determine the expression levels of PI3K, AKT, and mTOR in the messenger ribonucleic acid (mRNA).

**Results:**

There are 117 intersection targets of migraine and ED, involving 188 cell biological processes (BP), 21 cellular components (CC), 31 molecular functions (MF), and 65 signaling pathways. HE staining results show that there were no significant differences between Group A and Group B with regard to any of the parameters. Compared with Group A, the levels of the PI3K, p-AKT, and p-mTOR proteins and PI3K, AKT, and mTOR mRNAs in Group B decreased (*P* < 0.01).

**Conclusions:**

The decline of erectile function in a rat model of migraine was associated with the PI3K/Akt/mTOR signaling pathway.

## 1. Introduction

Migraine, one of the most common diseases of the primary central nervous system, predominantly manifests as a moderate-to-severe paroxysmal headache, but is often accompanied by nausea and vomiting [1]. The morbidity of migraine is relatively high; research has shown that 18% of females and 6% of males suffer from migraine [2]. The World Health Organization (WHO) ranks common chronic diseases according to the number of years lived with disability (YLD); migraine is in the top ten diseases when ranked in this manner. Moreover, severe migraine is considered to be the most disabling of the chronic diseases [3]. Erectile dysfunction (ED) is one of the most common forms of sexual dysfunction in males and can seriously affect both relationships and quality of life [4]. Over recent years, an increasing body of literature has focused on the specific relationship between ED and migraine. One cross-sectional study investigated 17,289 male patients with ED and showed that the risk of migraine in these patients was 1.69-folds higher than the risk for normal healthy males when excluding all other factors such as diabetes, hypertension, and cardiovascular disease [5].

However, the pathogenesis of migraine remains unclear. The mainstream view considers that the occurrence of migraine is related to the “trigeminal nerve-vascular theory” and the action of inflammatory pain-causing factors [6, 7]. The Phosphoinositide-3-kinase/protein kinase B/mechanistic target of rapamycin kinase (PI3K/Akt/mTOR) signaling pathway is an essential signaling pathway in the human body and facilitates the regulation of vascular endothelial proliferation, differentiation, and apoptosis [8]. Some studies have confirmed that PI3K and AKT undergo significant activation in brainstem tissue from rat models of migraine [9]. Furthermore, the levels of essential neurotransmitters in the brain, such as serotonin, were significantly reduced, thus proving that the PI3K/Akt/mTOR signaling pathway is one of the dominant mechanisms underlying migraine [10]. The occurrence of ED is due to a combination of factors, including blood vessels, nerves, and endocrine factors. PI3K, AKT, and other proteins are widely distributed on the surface of the penile vascular endothelium and smooth muscle; these factors play a significant role in vascular endothelial relaxation [11, 12], thought to be one of the key underlying causes of ED. However, whether migraine can affect erectile function via the PI3K/Akt/mTOR pathway remains unclear.

In the present study, we used bioinformatic technology to demonstrate that the PI3K/Akt/mTOR signaling pathway may represent a potential mechanism for the effects of migraine on erectile function. Next, we validated our bioinformatic results by carrying out in vivo animal experiments. Our primary aim was to identify the mechanism underlying the effect of migraine on ED.

## 2. Material and Methods

### 2.1. Drugs and Reagents

We purchased a range of reagents from GenePool (Beijing, China): sodium dodecyl sulfate- (SDS-) polyacrylamide gel electrophoresis (PAGE) Gel Kit (GPP1816), SDS-PAGE Loading Buffer (5x) (GPP1820), Protein Extraction Kit (GPP1815), Tris-Glycine Running Buffer (5x) (GPP1821), Total RNA Extraction Kit (DNase I) (GPQ1801), mRNA cDNA Synthesis Kit (GPQ1803), mRNA/lncRNA qPCR Kit (GPQ1808), and RNA Loading Buffer (5x) (GPQ181). Three different antibodies were used in this study: PI3K antibody (Abcam, ab191606; Cambridge, UK), p-AKT antibody (Bioss, bs-2720R; Beijing, China), and p-mTOR antibody (Bioss, bs-3494R; Beijing, China). We also used a range of key equipment, including an electrophoresis instrument (CAVOY, PP-1150), a spectrophotometer (NanoDrop 2000, Thermo Scientific), a fluorescence quantitative PCR instrument (LineGene 9600 Plus, Bioer Technology), and a double vertical electrophoresis tank (CAVOY, MP-8001).

### 2.2. Network Pharmacology Research

#### 2.2.1. The Identification of Molecular Targets

We identified the molecular targets for both migraine and ED by use of the GeneCards database (https://www.genecards.org/) and the Online Mendelian Inheritance in Man (OMIM) database (https://omim.org/). The separate targets for migraine and ED were then intersected, and the resultant targets that were associated with both disease states were considered as potential targets of the effects of migraine on ED. These targets were then used for further network construction and analysis.

#### 2.2.2. Protein-Protein Interaction (PPI) Network Analysis

The STRING database (https://string-db.org/) was used to identify PPIs. In order to improve the reliability of the data obtained, the PPIs were further filtered, the minimum interaction score was set to 0.40, and the remaining PPIs were used for network construction and analysis.

#### 2.2.3. Network Construction and Analysis

Cytoscape software (version 3.7.1, https://cytoscape.org/) was used to construct a migraine-ED-target network and a PPI network. The Cytoscape plugin “cytoHubba” was then used to analyze the PPI network to identify essential targets.

#### 2.2.4. Gene Ontology (GO) Term Analysis and Kyoto Encyclopedia of Genes and Genomes (KEGG) Enrichment Analysis

The biological information annotation database (DAVID, https://david.ncifcrf.gov/, version 6.8) provides systematic and comprehensive biological annotation of function for genes or proteins on a large scale and can then be used to identify the most significantly enriched biological annotations. We imported the most common targets for migraine and ED into the DAVID database, set the “identifier” as “‘Official Gene Symbol,” and set the “type list” to “Gene list.” We then performed GO terms and KEGG pathway enrichment analyses for the targets that were common to both migraine and ED.

### 2.3. Animal Experiment Research

#### 2.3.1. Experimental Animals

For the in vivo experiments, we purchased 20 Sprague-Dawley rats (specific pathogen free [SPF], 6 weeks of age, weight range: 200-210 g) (Authorization number: SCXK [Beijing] 2016-0006) from Beijing Vital River Laboratory Animal Technology Co., Ltd. The rats were kept in a SPF animal house in Dongzhimen Hospital affiliated to the Beijing University of Chinese Medicine. The photoperiod of the animal holding environment was 12 hours light/12 hours dark; humidity was 55%-60%, and the temperature ranged from 22 to 26°C. All rats were allowed to feed on a solid diet and drink deionized water ad libitum for seven days prior to the experiment. Before the experiment, we carried out mating behavior tests and noncontact penile erection tests to confirm that all rats showed normal levels of sexual function. All experimental protocols conformed to the guidelines approved by the Animal Ethics Committee of Dongzhimen Hospital affiliated to Beijing University of Chinese Medicine (Approval Number: 20-27).

#### 2.3.2. Animal Grouping and Modeling

Twenty male SD rats were randomly divided into a blank group (Group A, *n* = 10) and a migraine model group (Group B, *n* = 10). The rats in Group A were subcutaneously injected with normal saline (2 ml/kg) into the back of the neck. Rats in Group B were subcutaneously injected with nitroglycerin 10 mg/kg (5 mg/ml) into the back of the neck in order to create an animal model of migraine.

#### 2.3.3. Validating the Model of Migraine

Rats were placed in a transparent observation box immediately after modeling. From the beginning of modeling, we observed the general status of the experimental rats and counted the number of times they scratched their heads and climbed their cage, at different time points after modeling. The rats in Group B showed increased levels of head scratching with their forelimbs; they also climbed their cages frequently. This type of behavior is a typical characteristic of migraine, along with fatigue and curling up. Since we observed these characteristics in our rats, we were able to confirm that we had successfully created a rat model of migraine.

#### 2.3.4. Determination of Erectile Function

We determined erectile function using the method previously described by Hong et al. [13]. Two groups of rats were weighed and placed in a quiet and dim observation box for 10 minutes to adapt. Next, a preparation of apomorphine (100 *μ*g/kg) was subcutaneously injected into the neck. We then used a camera to take photographs of each rat within its environment for a total of 30 minutes. During this time period, we monitored the erectile condition of the penis of each rat. An erection was defined as when the penis became expanded or enlarged and when the end of the penis became exposed.

#### 2.3.5. Preparation of Tissue Samples

Two hours after successful modeling, the rats were weighed and anesthetized with 1% sodium pentobarbital (50 mg/kg) intraperitoneally. Next, we removed the penis from each rat for protein extraction and western blotting, Reverse Transcription-Polymerase Chain Reaction (RT-qPCR), and morphological analyses.

### 2.4. Hematoxylin and Eosin Staining

Penile tissue was first cleaned; one half of the tissue was embedded in paraffin wax and cut into 5 *μ*m sections. These sections were stained in hematoxylin-eosin (HE) and observed by light microscopy. Images were acquired using an optical microscope equipped with a digital tube (Olympus BX51TF, Tokyo, Japan).

### 2.5. Western Blotting

Total protein was extracted from each of the penis tissue samples. The concentration of protein in each tissue sample was determined with a bicinchoninic acid protein assay kit (BCA kit). Protein samples were then separated by SDS-PAGE, transferred to a polyvinylidene fluoride (PVDF) membrane, and finally incubated overnight at 4°C. The next morning, the membrane was washed with PBS, incubated with a secondary antibody for 30 min, and then rewashed. Positive binding was visualized by the application of an ECL developer. Next, we used Quantity One software v.4.6.2 (Bio-Rad, Hercules, California, USA) to analyze the optical density of each protein band.

### 2.6. RT-qPCR

RT-qPCR detection was carried out using a PCR machine (slan-96p; Shanghai Hongshi, Shanghai, China). Total RNA was extracted from penile tissue using the TRIzol kit. Next, we determined the purity and concentration of the total RNA and prepared cDNA by reverse transcription. The primer sequences are shown in Table 1. Samples were added to a 96-well plate and fluorescence quantitative PCR was carried out to amplify target DNA fragments. We recorded the number of cycle times (Ct value) for the fluorescent signal once the set threshold had been reached. We then determined the differences in gene expression by comparing the multiple of the target gene relative to the reference gene with the multiple of the target gene using the relative quantitative (RQ) method (RQ = 2^−CtΔΔ^) [14].

### 2.7. Statistical Analysis

All analyses will be performed using SPSS software (version 26.0, Armonk, NY, USA), with a two-sided *P* value < 0.05 considered significant. Continuous variables will be compared using the *t*-test or Wilcoxon rank-sum test as appropriate. Since the indicators we collect are all measurement data, the experiment is randomly grouped. After testing, the normality test was *P* > 0.05 and the variance homogeneity test *P* > 0.05, so the independent sample *t*-test was used.

## 3. Results

### 3.1. Network Pharmacology Research

#### 3.1.1. Target Collection

**Figure 1:**
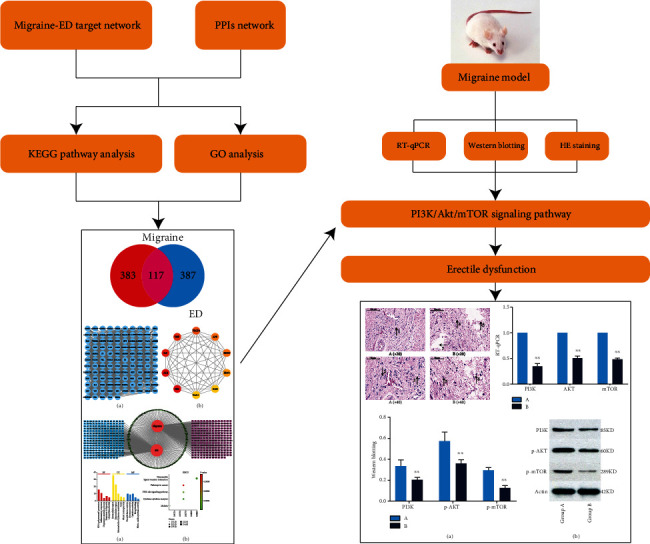
Study flowchart. We predicted the proteins and pathways associated with migraine and erectile dysfunction. We used RT-qPCR and western blotting to measure expression of proteins and mRNA in rat penis cells of each group. The results of in vivo experiments were consistent with the prediction results of bioinformatic analysis. The decline of erectile function in a rat model of migraine was associated with the PI3K/Akt/mTOR signaling pathway.

**Figure 2:**
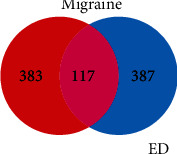
Venn diagram: intersection of targets for migraine and targets for erectile dysfunction. We predicted the intersection of targets for erectile dysfunction and targets for migraine. Individual targets for erectile dysfunction are shown as a blue circle. Individual targets for migraine are shown as a red circle. The intersection of these targets is shown as a purple circle.

Figure 1 shows a flowchart depicting the process underlying our research. GeneCards screening allowed us to identify 500 and 500 targets for migraine and ED, respectively. Following intersection, we identified 117 targets that were related to both migraine and ED (Figure 2).

#### 3.1.2. Construction and Topological Analysis of a Migraine-ED Network and PPI Network

**Figure 3:**
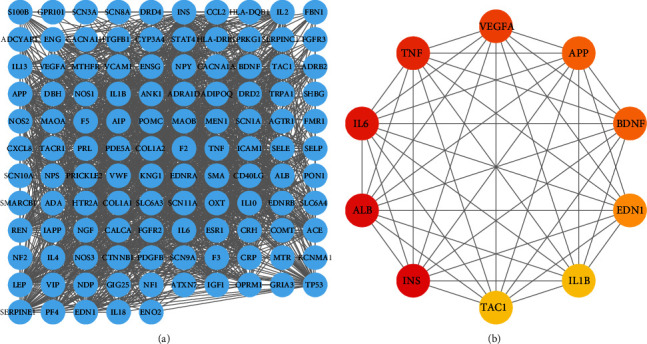
PPI network: (a) PPI network built by Cytoscape v3.7.1; (b) PPI network processed by a Cytoscape v3.7.1 plugin called cytoHubba. The node color is proportional to the degree of PPI.

**Figure 4:**
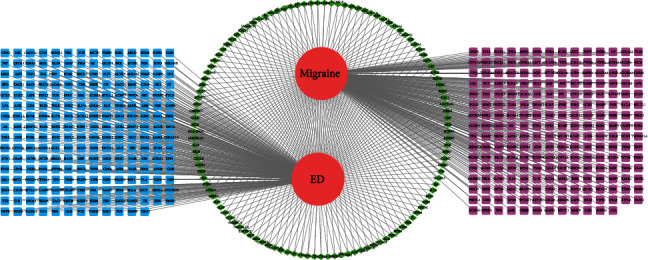
Network construction for migraine-erectile dysfunction. Red circle: migraine and erectile dysfunction; purple square: migraine individual targets; blue square: erectile dysfunction individual targets; green diamond: intersecting targets.

Next, the 117 intersecting targets for migraine and ED were analyzed with the STRING database. Cytoscape software was used to construct a PPI network for the 117 targets (Figure 3(a)). The cytoHubba plugin was then used to analyze the PPI network, and the top five degrees were regarded as key targets: insulin (INS) (ranked 1), albumin (ALB) (ranked 2), interleukin 6 (IL6) (ranked 3), tumor necrosis factor (TNF) (ranked 4), and vascular endothelial growth factor A (VEGFA) (ranked 5) (Figure 3(b) and Table 2). Cytoscape software was also used to construct a network featuring migraine and ED (Figure 4).

#### 3.1.3. GO Biological Process Enrichment Analysis

**Figure 5:**
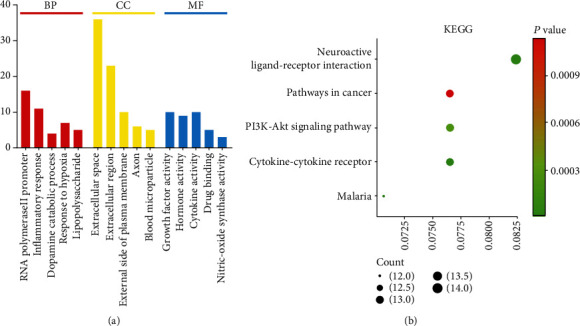
GO enrichment analysis and KEGG enrichment analysis. (a) The *X* axis is the top five results of BP, CC, and MF *P* values. The *Y* axis is the number of enriched targets (count). red bar: BP; yellow bar: CC; blue bar: MF. (b) *Y* axis is the name. *X* axis is the richness factor. The size of the node is proportional to the number of genes. The node color is proportional to the *P* value.

DAVID version 6.8 was used to carry out enrichment analyses on the 117 targets; this research showed that these targets were associated with 188 cell biological processes (BP), 21 cellular components (CC), 31 molecular functions (MF), and 65 signaling pathways. Next, we selected the top five biological functions and signal pathways, as determined by *P* values (Figure 5).

### 3.2. Animal Experiment Research

#### 3.2.1. Validation of a Rat Model of Migraine

Compared with Group A, the rats in Group B showed red ears, frequent episodes of head scratching, an increased incidence of cage climbing, and an increased incidence of back-and-forth movement; these events were observed just 5–10 minutes after modeling began. Approximately 120 minutes later, the rats displayed a lack of energy, slower responses, and reduced levels of activity (Table 3).

#### 3.2.2. Erection Times

We monitored the number of erections occurring in each group of rats for 30 minutes after the injection of apomorphine. When compared to Group A, we found that there was a significantly lower number of erections in Group B (*P* < 0.01) (Table 4).

### 3.3. HE Staining of Penile Tissue

**Figure 6:**
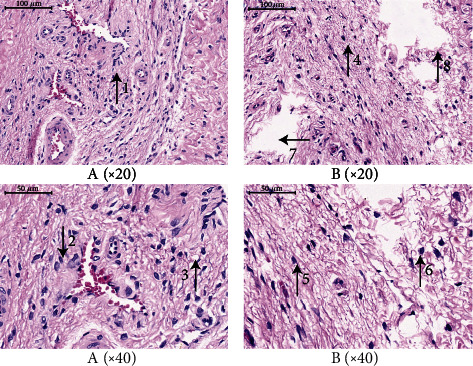
Analyses of penis tissue in rats using HE staining. The penis tissue of rats was stained with hematoxylin and eosin (HE) to observe pathologic changes in the penis under electron microscopy (*n* = 10 animals per group). In the two Groups A and B, the cavernous trabeculae are evenly distributed, with abundant nuclei, small tissue gap, and no obvious fibrous proliferation (arrows 1–6). The parts shown by arrows 7 and 8 are due to tissue tearing or shedding caused during the operation, not pathological tissue gap increase. Therefore, there is no difference in the analysis of HE tissue staining between the A and B groups.

Next, we used light microscopy to investigate HE-stained sections of penile tissue. The corpus cavernosum of both Group A and Group B showed cavernous trabeculae and an even distribution of blood sinusoids. Some red blood cells were seen in the sinus space, and the inner wall of the blood sinusoid was covered with endothelial cells. The sinusoid trabeculae contained a large quantity of smooth muscle, collagen fibers, and some small blood vessels; there was no proliferation of the interstitial tissue. There were no significant differences between Group A and Group B with regard to any of the parameters (Figure 6).

### 3.4. The Expression Levels of PI3K, p-AKT, and p-mTOR Proteins in Rat Penile Tissues

**Figure 7:**
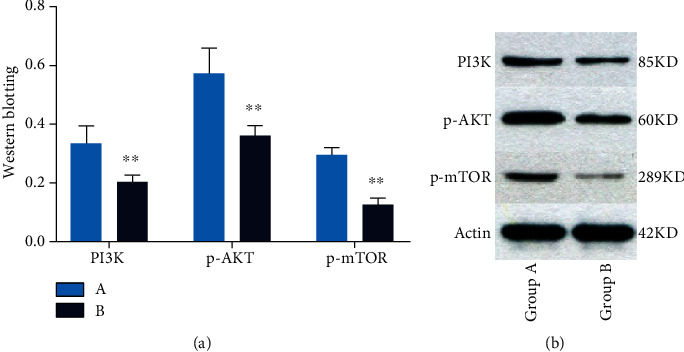
Expression of PI3K, p-AKT, and p-mTOR in rat penis. (a) The light blue chart represents expression of PI3K, p-AKT, and p-mTOR in Group A. The navy blue chart represents expression of PI3K, p-AKT, and p-mTOR in Group B. Values are the mean ± SEM (*n* = 10 animals per group). Student's *t*-test was used. Group B was compared with Group A, ^∗^*P* < 0.05 and ^∗∗^*P* < 0.01. (b) Western blotting showing expression of PI3K, p-AKT, and p-mTOR proteins. Actin is a loading control.

Western blotting was used to detect the expression of several key proteins in the penile tissue of rats in each group. By analyzing the gray value of protein bands, we were able to ascertain that the level of PI3K protein was significantly lower in rats from Group B when compared to Group A (*P* < 0.01). Compared with Group A, the expression levels of the p-AKT protein in rats from Group B were significantly reduced (*P* < 0.01). Compared with Group A, the expression levels of the p-mTOR protein in rats from Group B were significantly reduced (*P* < 0.01) (Figure 7).

### 3.5. Expression Levels of PI3K, AKT, and mTOR mRNAs in Rat Penile Tissues

**Figure 8:**
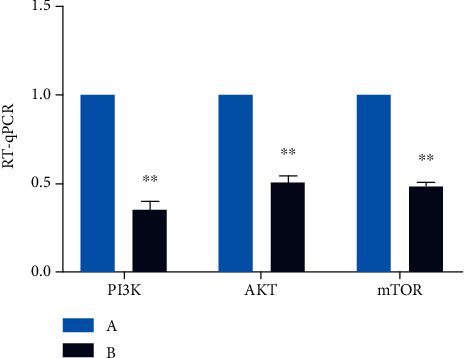
Expression of the mRNA of PI3K, AKT, and mTOR in rat penis. The light blue chart represents expression of the mRNA of PI3K, AKT, and mTOR in Group A. The navy blue chart represents expression of the mRNA of PI3K, AKT, and mTOR in Group B. Values are the mean ± SEM (*n* = 10 animals per group). Student's *t*-test was used. Group B was compared with Group A, ^∗^*P* < 0.05 and ^∗∗^*P* < 0.01.

Next, we used RT-qPCR to analyze the expression levels of mRNA between the two groups. Compared to Group A, the levels of the PI3K mRNA in Group B were significantly reduced (*P* < 0.01). Compared to Group A, the expression level of the AKT mRNA in Group B was significantly reduced (*P* < 0.01); comparing with Group A, the expression levels of the mTOR mRNA in Group B were significantly reduced (*P* < 0.01) (Figure 8). Collectively, these results identified significant changes in the expression levels of mRNAs related to the PI3K pathway in penile tissue acquired from rats experiencing migraine.

## 4. Discussion

Migraine is one of the most common diseases of the primary central nervous system. Chronic and recurring migraine can lead to a decline in daily work and living ability, a reduction in working hours, or even the inability to perform work; these effects can have severe impact on health and social function [15, 16]. Males who suffer from migraine, especially those who suffer from severe forms of migraine, can also suffer from a decline in erectile function; however, the specific molecular mechanisms associated with these effects have yet to be fully elucidated. In the present study, we mined a range of biological data and discovered that the association between migraine and ED might be related to the PI3K/AKT/mTOR pathway. We validated this using in vivo experiments in a rat model of migraine and demonstrated that in vivo data were consistent with those derived from biological prediction.

The PI3K/AKT/mTOR signaling pathway is an essential signaling pathway in the human body. PI3K is an intracellular phosphatidylinositol kinase that exhibits lipid kinase activity and protein kinase activity. The inflammatory signal molecules produced during migraine can bind to cell surface receptors, thereby promoting the activation of PI3K. Activated PI3K is subsequently phosphorylated on the lipid membrane to form phosphatidylinositol 3,4,5-triphosphate (PIP3) [17, 18]. PIP3 can then bind to intracellular signal protein filaments or the pleckstrin homology (PH) domain of threonine protein kinase/protein kinase B (AKT/PKB) and PDK1 (phosphoinositide-dependent kinase-1) to promote the phosphorylation of AKT, which in turn causes the phosphorylation of mTOR [19]. The activation of mTOR allows the formation of the mTOR complex 1 (TORC1) which is known to affect vascular endothelial function [20].

In addition to the regulation of blood vessels, nerves, the endocrine system, and other factors, abnormalities in penile erection can also be closely related to the vasomotor function of the vascular smooth muscle. Previous studies have found that during the occurrence of ED, there is an increase in the number of collagen fibers in the penile cavernous body; fibrotic changes can also occur, along with limitations in smooth muscle relaxation and contraction [10]. The PI3K/Akt/mTOR pathway is widely distributed in the penile vascular smooth muscle and facilitates the regulation of diastolic function in the smooth muscle. Numerous adverse factors, such as a high glucose environment, oxidative stress, and inflammatory responses, can activate the PI3K pathway to induce the phosphorylation of AKT, thus promoting protein expression downstream [21–23]. mTOR is an essential protein that regulates the cell metabolism and autophagy. The activation of mTOR can significantly inhibit the level of autophagy and thus prolong cell lifespan [21, 24]. Huang et al. [25] reported that low-dose shock waves, combined with mesenchymal stem cells, regulate the smooth muscle via the PI3K/Akt/mTOR pathway, thus exhibiting a better therapeutic effect for ED.

Huang et al. found that according to an epidemiological survey of 5763 ED patients in Taiwan, among ED patients, the odds of having been previously diagnosed with migraines was 1.63 (95% CI, 1.39-1.91). This risk was more pronounced in younger groups, with the highest risk being detected among those aged between 30 and 39 years [26]. This is also consistent with the results of our study, which proves the effect of migraine on erectile function from a clinical perspective. However, whether the mechanism in the human body is related to the PI3K/Akt/mTOR pathway has not been reported.

Psychological effects may be used as an intermediate link, that is, migraine affects psychological factors, which in turn lead to erectile dysfunction. Migraine is a common chronic painful disease that involves many people in the world and has various degrees of mental and psychological problems, often accompanied by anxiety and depression. Many neuropsychiatric diseases are closely related to many chronic or systemic diseases, which may induce sexual dysfunction [29]. Therefore, we cannot completely exclude ED caused by psychological factors. However, our article, as the first experimental study to report the correlation between migraine and ED, only made a preliminary exploration and found that the PI3K/Akt/mTOR pathway related to erectile dysfunction has undergone significant changes, which proves that migraine does have a direct or indirect effect on erectile function.

In this study, we found that the expression levels of PI3K, p-AKT, and p-mTOR proteins and PI3K, Akt, and mTOR mRNAs in the penile tissue of rats that experienced migraine were significantly reduced. Therefore, we believe that the decline of erectile function in rats experiencing migraine is closely related to the PI3K/Akt/mTOR signaling pathway. This study had certain limitations that need to be considered. For example, the levels of sex hormones, especially testosterone, are known to exert specific influence on erectile function. In the present study, we used bioinformatic analysis to compare key proteins. Our bioinformatic prediction did not include indicators associated with sex hormone levels. Due to funding constraints, we selected the PI3K/Akt/mTOR as the entry point for our research. Future research should attempt to investigate changes in the levels of serum sex hormones in rat models of migraine experiencing ED. In addition, we cannot rule out whether psychological factors are involved in the erectile dysfunction caused by migraine as an intermediate link. Based on this, we will follow-up cross-sectional research combined with experimental research to further verify whether psychological factors are involved in the above process.

## 5. Conclusion

Our analyses found that the decline of erectile function in a rat model of migraine was associated with the PI3K/Akt/mTOR signaling pathway. Furthermore, we found that migraine can inhibit the expression of key proteins and mRNAs in the PI3K/Akt/mTOR pathway, and these factors together lead to the decline of erectile function.

## Figures and Tables

**Table 1:** Specific primer information. Primers were purchased from Invitrogen Biotechnology Co. Ltd. (SJ, CN) and tested by Beijing Jipu Biotechnology Co., Ltd.

Primer		Primer sequence (5′ to 3′)
PI3K	Upstream	AATGATGCTTGGCTCTGGAATG
	Downstream	TGCTGCTTGATGGTGTGGAA
AKT	Upstream	ATGAACGACGTAGCCATTGTG
	Downstream	TTGTAGCCAATAAAGGTGCCAT
mTOR	Upstream	TTCAATCCATAGCCCCGTCT
	Downstream	CAAAGAGCTGCATCACTCGT
Actin	Upstream	GCCTTCCTTCTTGGGTAT
	Downstream	GGCATAGAGGTCTTTACGG

**Table 2:** Information relating to the five key target genes, as determined by the cytoHubba plugin in Cytoscape software.

Rank	Gene name	Score (degree)
1	*INS*	80
2	*ALB*	76
3	*IL6*	75
4	*TNF*	66
5	*VEGFA*	64

**Table 3:** Observation index.

	Number	Red ears	Frequent episodes of head scratching	Cage climbing	Back-and-forth movement	Lack of energy	Slower responses
Group A	10	—	—	—	—	—	—
Group B	10	+	++	++	++	+++	++

“+” denotes the degree: the more “+,” the greater is the degree.

**Table 4:** The number of erections (*x* ± *s*, *n* = 10) of rats in each group.

Group	Number of erections
A	3.8 ± 1.03
B	0.9 ± 0.74^∗∗^

The number of erections in thirty minutes was observed by injecting APO into the neck at a dose of 100 *μ*g/kg. Group B was compared with Group A, ^∗∗^*P* < 0.01.

## Data Availability

The data used to support the findings of this study are available from the corresponding authors upon request.

## References

[1] May A., Schulte L. H. (2016). Chronic migraine: risk factors, mechanisms and treatment. *Nature reviews. Neurology*.

[2] Grøtta V. K., MacGregor E. A. (2017). Sex differences in the epidemiology, clinical features, and pathophysiology of migraine. *The Lancet. Neurology*.

[3] Leonardi M., Steiner T. J., Scher A. T., Lipton R. B. (2005). The global burden of migraine: measuring disability in headache disorders with WHO's classification of functioning, disability and health (ICF). *The journal of headache and pain*.

[4] Burnett A. L., Nehra A., Breau R. H. (2018). Erectile dysfunction: AUA guideline. *The Journal of urology*.

[5] Wu S.-H., Chuang E., Chuang T.-Y. (2016). A nationwide population-based cohort study of migraine and organic-psychogenic erectile dysfunction. *Medicine*.

[6] Charles A. (2018). The pathophysiology of migraine: implications for clinical management. *The Lancet. Neurology*.

[7] Ashina M., Hansen J. M., Do T. P., Melo-Carrillo A., Burstein R., Moskowitz M. A. (2019). Migraine and the trigeminovascular system-40 years and counting. *The Lancet. Neurology*.

[8] LoRusso P. M. (2016). Inhibition of the PI3K/AKT/mTOR pathway in solid tumors. *Journal of clinical oncology*.

[9] Rodon J., Dienstmann R., Serra V., Tabernero J. (2013). Development of PI3K inhibitors: lessons learned from early clinical trials. *Nature reviews. Clinical oncology*.

[10] Luan Y., Cui K., Tang Z. (2020). Human Tissue Kallikrein 1 Improves Erectile Dysfunction of Streptozotocin- Induced Diabetic Rats by Inhibition of Excessive Oxidative Stress and Activation of the PI3K/AKT/eNOS Pathway. *Oxidative medicine and cellular longevity*.

[11] Hui J., Liu R., Zhang H., He S., Wei A. (2020). Screening and identification of critical biomarkers in erectile dysfunction: evidence from bioinformatic analysis. *PeerJ*.

[12] Heaton J. P. W., Varrin S. J., Morales A. (1991). The characterization of a bio-assay of erectile function in a rat model. *The Journal of urology*.

[13] Hong K. M., Najjar H., Hawley M., Press R. D. (2004). Quantitative real-time PCR with automated sample preparation for diagnosis and monitoring of cytomegalovirus infection in bone marrow transplant patients. *Clinical chemistry*.

[14] Steiner T. J., Stovner L. J., Birbeck G. L. (2013). Migraine: the seventh disabler. *Cephalalgia*.

[15] Martelletti P., Schwedt T. J., Lanteri-Minet M. (2018). My migraine voice survey: a global study of disease burden among individuals with migraine for whom preventive treatments have failed. *The journal of headache and pain*.

[16] Xie Y., Shi X., Sheng K. (2018). PI3K/Akt signaling transduction pathway, erythropoiesis and glycolysis in hypoxia (review). *Molecular medicine reports*.

[17] Toren P., Zoubeidi A. (2014). Targeting the PI3K/Akt pathway in prostate cancer: challenges and opportunities (review). *International journal of oncology*.

[18] Zhang Y., Yan H., Xu Z., Yang B., Luo P., He Q. (2019). Molecular basis for class side effects associated with PI3K/AKT/mTOR pathway inhibitors. *Expert opinion on drug metabolism & toxicology*.

[19] Barra F., Evangelisti G., Ferro Desideri L. (2019). Investigational PI3K/AKT/mTOR inhibitors in development for endometrial cancer. *Expert opinion on investigational drugs*.

[20] Shamloul R., Ghanem H. (2013). Erectile dysfunction. *The Lancet*.

[21] Zhu G. Q., Jeon S. H., Bae W. J. (2018). Efficient promotion of autophagy and angiogenesis using mesenchymal stem cell therapy enhanced by the low-energy shock waves in the treatment of erectile dysfunction. *Stem cells international*.

[22] Tang Z., Cui K., Luan Y. (2018). Human tissue kallikrein 1 ameliorates erectile function via modulation of macroautophagy in aged transgenic rats. *Andrology*.

[23] Zhang J., Wu X.-J., Zhuo D.-X. (2010). Effect of tankyrase 1 on autophagy in the corpus cavernosum smooth muscle cells from ageing rats with erectile dysfunction and its potential mechanism. *Asian journal of andrology*.

[24] Ding J., Tang Y., Tang Z. (2018). Icariin improves the sexual function of male mice through the PI3K/AKT/eNOS/NO signalling pathway. *Andrologia*.

[25] Huang C.-Y., Keller J. J., Sheu J.-J., Lin H.-C. (2012). Migraine and erectile dysfunction: evidence from a population-based case-control study. *Cephalalgia*.

[26] Aksoy D., Solmaz V., Cevik B., Gencten Y., Erdemir F., Kurt S. G. (2013). The evaluation of sexual dysfunction in male patients with migraine and tension type headache. *The journal of headache and pain*.

